# Cardioprotective role of APIP in myocardial infarction through ADORA2B

**DOI:** 10.1038/s41419-019-1746-3

**Published:** 2019-07-01

**Authors:** Bitna Lim, Kwangmin Jung, Youngdae Gwon, Jae Gyun Oh, Jae-il Roh, Se-Hoon Hong, Changwon Kho, Woo-Jin Park, Han-Woong Lee, Jang-Whan Bae, Yong-Keun Jung

**Affiliations:** 1 School of Biological Science, Seoul National University, Seoul, 08826 Korea; 2 Cardiovascular Research Center, Icahn School of Medicine at Mount Sinai, New York, NY 10029 USA; 3 Department of Life Science, Gwangju Institute of Science and Technology, Gwangju, 61005 Korea; 4 Department of Biochemistry, Yonsei University, Seoul, 03722 Korea; 5 Department of Internal Medicine, Chungbuk National University College of Medicine, Cheongju, 28644 Korea

**Keywords:** Apoptosis, Circulation

## Abstract

In ischemic human hearts, the induction of adenosine receptor A2B (ADORA2B) is associated with cardioprotection against ischemic heart damage, but the mechanism underlying this association remains unclear. Apaf-1-interacting protein (*APIP)* and *ADORA2B* transcript levels in human hearts are substantially higher in patients with heart failure than in controls. Interestingly, the *APIP* and *ADORA2B* mRNA levels are highly correlated with each other (*R* = 0.912). APIP expression was significantly increased in primary neonatal cardiomyocytes under hypoxic conditions and this induction reduced myocardial cell death via the activation of the AKT-HIF1α pathway. Accordingly, infarct sizes of APIP transgenic mice after left anterior descending artery ligation were significantly reduced compared to those of wild-type mice. Strikingly, knockdown of APIP expression impaired the cytoprotective effects of ADORA2B during hypoxic damage. Immunoprecipitation and proximity ligation assays revealed that APIP interacts with ADORA2B, leading to the stabilization of both proteins by interfering with lysosomal degradation, and to the activation of the downstream PKA-CREB signaling pathways. ADORA2B levels in the hearts of *APIP*^Tg/Tg^, *APIP*^Tg/+^, and *Apip*^+/-^ mice were proportionally downregulated. In addition, ADORA2B D296G derived from the rs200741295 polymorphism failed to bind to APIP and did not exert cardioprotective activity during hypoxia. Moreover, *Adora2b* D296G knock-in mice were more vulnerable than control mice to myocardial infarction and intentional increases in APIP levels overcame the defective protection of the ADORA2B SNP against ischemic injury. Collectively, APIP is crucial for cardioprotection against myocardial infarction by virtue of binding to and stabilizing ADORA2B, thereby dampening ischemic heart injury.

## Introduction

Myocardial infarction (MI) is one of the main causes of mortality and morbidity worldwide^1^. Once MI occurs, various types of cell death, such as necrosis, apoptosis, or autophagy-associated cell death, are activated^2^. The generation of multiple dying cells that are highly immunostimulatory during ischemic injury causes the infiltration of inflammatory leukocytes and production of various cytokines^2,3^. Through feed-forward cycles, a continuous inflammatory response leads to myocardial apoptosis, which triggers the onset of heart failure^4–6^. In the absence of an effective treatment for MI, further research is necessary to evaluate anti-apoptotic and anti-inflammatory strategies.

Since adenosine signaling was initially proposed in the 1970s, four subtypes of adenosine receptors, A_1_, A_2A_, A_2B_, and A_3_, have been reported^7,8^. They are expressed in various organs and are regulated in a variety of diseases including inflammation, cancer, and glaucoma^9^. The function of the adenosine A_2B_ receptor (ADORA2B) in pathological conditions has attracted substantial attention owing to its lower expression in most tissues compared to that of the other receptor subtypes, combined with its low affinity for adenosine^8,10^. When the extracellular adenosine concentration is increased in hypoxia, cytoprotective signaling from ADORA2B begins in the kidney, intestine, lung, heart, and other organs^10–12^. However, genetic modifiers that directly regulate ADORA2B in damaged cardiomyocytes have not been identified.

Apaf-1-interacting protein (APIP) suppresses various types of cell death, such as caspase-9-dependent apoptosis and caspase-1-dependent pyroptosis^13,14^. In addition, SNPs near *APIP* have been found in patients with systemic inflammatory response syndrome^14^. Intriguingly, APIP also has 5-methylthioribulose-1-phosphate dehydratase (MtnB) activity in the methionine salvage pathway^15^. The function of APIP as an MtnB is independent of its ability to inhibit apoptosis but is related to caspase-1-induced pyroptosis^15^. While APIP is ubiquitously expressed in most human adult tissues, it is highly expressed in the skeletal muscle and heart^13^. Previously, we demonstrated that APIP inhibits hypoxic cell death by inducing sustained activation of AKT and ERK1/2 in muscle cells, but the mechanism underlying this protective effect is unknown^16^.

In this study, we observed that APIP is crucial for cardioprotection under hypoxia. In particular, APIP stimulates adenosine receptor signaling via its interaction with ADORA2B upon ischemic injury and overcomes the defective protection of the ADORA2B SNP against ischemic injury in vitro and in vivo, providing a mechanistic explanation for the cardioprotective effects of ADORA2B against MI and a rationale for the therapeutic potential of APIP in MI.

## Methods and Materials

### Generation of transgenic, knockout and knockin mice

*APIP* transgenic (*APIP*^Tg/+^) and *Apip* knockout (*Apip*^+/−^) mice were generated as previously described^17^. *Adora2b* D296G^KI/KI^ mice were generated by Dr. HW Lee (Yonsei University, Korea) and the mice were interbred and maintained in a pathogen-free condition. All animal experiments were performed in accordance with the Korean Food and Drug Administration (KFDA) guidelines. Briefly, C57BL/6 N female mice were treated with PMSG and HCG and mated with C57BL/6 N stud male mice after 48 h. Next day, fertilized embryos were harvested from female mice and microinjected with sgRNA and Cas9 protein mixture. Knock-in mice were produced by the standard method and identified by PCR analysis of the mouse tail DNA using the primer pairs for the *Adora2b* gene. For each group, only male mice were used (8–12 weeks of age). Gene-targeted, transgenic and littermate control mice were matched in age, gender and weight. All animals used in this study were maintained under the animal protocols of Seoul National University guidelines and the study was approved by the Animal Care and Use Committee at Seoul National University (SNU-161117–2–1, SNU-180328-2).

### Cell culture

H9c2, HEK293T, and HeLa cells were cultured in Dulbecco’s Modified Eagles Medium (DMEM, HyClone) supplemented with 10% fetal bovine serum (FBS, Gibco) in humid air with 5% CO_2_ at 37 °C. Neonatal cardiomyocytes were isolated from 1- to 2-day-old mice as previously described^18^. Briefly, neonatal mouse ventricles were dispersed on a series of incubations at 37 °C in the buffer containing 2 mg/ml collagenase type II (Worthington Biochemical Corp.). The supernatant was collected and centrifuged at 200 × *g* for 8 min and the cells were resuspended and cultured in DMEM with 20% FBS in humid air with 5% CO_2_ at 37 °C. To exclude non-muscle cells, cells in the supernatant were isolated for further culture after 60 min of incubation, while adherent cells were discarded. Experiments were performed between days 2 and 3 in culture.

### Surgery of Left Anterior Descending Coronary Artery Ligation

Induction of MI was performed in 8- to 12-weeks-old mice as described previously^19,20^. Briefly, mice were anesthetized by *i.p*. injection with a mixture of Zoletil and Rompun. Mice were incubated with a 22-gauge intravenous catheter and ventilated with a rodent ventilator (Harvard Apparatus, Holliston, MA, USA). A Left thoracotomy was performed at the fourth intercostal space and the LAD was identified and ligated at ~2 mm from the tip of the left auricle using a 7–0 silk suture. Ligation success was confirmed when the anterior wall of the left ventricle turned pale. The chest cavity was closed using 5–0 suture and the animal was allowed to recover on the heat pad. The body temperature was continuously maintained between 35 ~36 °C throughout the procedure. Dead mice within 2–3 h after surgery were excluded from the further analysis.

### Echocardiography

Transthoracic echocardiography was performed as described^21^. The mice were anesthetized with avertin (250 mg/kg) to measure cardiac function. Transthoracic echocardiography was performed using a Powervision 6000 (TOSHIBA) instrument and a 12 MHz microprobe (PLM-1204AT, TOSHIBA). The M-mode measurements of the left ventricle were obtained on the long-axis view of the two-dimensional mode.

### Hypoxia protocol

Neonatal cardiomyocytes or H9c2 cells were placed in a modular incubator chamber and were flushed with the gas mixture of 1% O_2_, 5% CO_2_ and 94% N_2_ for minimum 10 min. After that, the sealed chamber was placed in a 37 °C ℃ incubator for the indicated times. To observe regulation of HIF1α, hypoxia was established for 2–6 h, cell death was observed for at least 18 h of hypoxia.

### Proximity Ligation Assay

Protein interactions were detected by Duolink *In Situ* reagents (Sigma, St. Louis, MO, USA) according to the supplier’s protocol. Briefly, the cells were fixed in 4% paraformaldehyde, permeabilized in 0.1% Triton X-100 for 10 min at room temperature and then incubated with primary antibodies: mouse anti-APIP (1:100 dilution) and rabbit anti-ADORA2B (1:100 dilution) at 4 °C overnight. The samples were incubated with secondary antibodies conjugated with PLA probes for 1 h at 37 °C, followed by hybridization and ligation reaction. Rolling-circle amplification was then performed to generate concatemeric fluorescent products which were visualized as distinct fluorescent dots under a Zeiss LSM 700 confocal microscope (Carl Zeiss).

### DNA transfection and reagents

Transfection was carried out using Polyfect® reagent (Qiagen, Valencia, CA), Lipofectamine® (Invitrogen) or PEI (Sigma-Aldrich) according to the manufacturers’ instructions. BAY60-6583 was purchased from R&D systems (Minneapolis, MN) and 5′‐*N*‐ethylcarboxamidoadenosine (NECA) was obtained from EMD Millipore.

### Cell Death and Cell Proliferation Assays

To determine cell death, cells were analyzed with trypan blue exclusion method or assessed by counting GFP-positive cells showing apoptotic fractured nuclei characterized by ethidium homodimer-1 (Molecular Probe). Cell viability was also determined using the EZ-CyTox cell viability kit (Daeil Lab Service, South Korea) according to the manufacturer’s protocol.

### Western blotting

Cultured cells or heart tissues were lysed with RIPA (50 mM Tris-Cl pH 8.0, 15 mM NaCl, 1% Triton X-100, 0.5% sodium deoxycholate, 0.1% SDS, 1 mM PMSF, and 1 mg/ml each of aprotinin, leupeptin and pepstatin A) or CHAPS (150 mM NaCl, 1% CHAPS, 2 mM EDTA, and 25 mM HEPES pH 7.4) lysis buffer. Equal amounts of proteins were electrophoresed on 8 to 15% gel by sodium dodecyl sulfate-polyacrylamide gel electrophoresis and transferred to polyvinylidene difluoride membranes by semi-dry transfer system (BioRad). The membranes were blocked with 3% BSA in Tris-buffered saline and 0.2% Tween (TBST) at room temperature for 1 h and then incubated overnight at 4 °C or at room temperature for 2 h with the following specific primary antibodies: anti-APIP (NB100–56567, Novus Biologicals), anti-ADORA2B (LS-B12113, LifeSpan BioSciences), anti-ADORA3 (ab203298, Abcam), anti-pAKT (Ser473) (9271, Cell signaling), anti-AKT (9272, Cell signaling), β-actin (sc-47778, Santa Cruz Biotech), and α-tubulin (sc-23948, Santa Cruz Biotech). All the primary antibodies were diluted by 1,000 to 3,000 times when used for immunoblotting. The blots were then washed with TBST and incubated with secondary antibodies for 60 min at room temperature. Protein expression levels were quantified by scanning densitometries and normalized by those of β-actin or α-tubulin.

### Immunoprecipitation

Cell extracts were solubilized in RIPA lysis buffer and then incubated for 3 h at 4 °C with immunoglobulin G (IgG) or anti-HA, anti-FLAG (Sigma Aldrich) and anti-APIP antibodies. The mixtures were further incubated for an additional 3 h at 4 °C after adding another 30 μl protein G sepharose^TM^ (GE Healthcare). The immunocomplexes were then collected by centrifugation at 5,000 r.p.m. for 3 min, washed 3 times with lysis buffer and detected by western blotting.

### Subcellular Fractionation

Cell lysates were homogenized in hypotonic buffer (250 mM Sucrose, 20 mM HEPES pH 7.4, 10 mM KCl, 1.5 mM MgCl_2_, and 1 mM EDTA) by passing through a 26-gauge needle. Nuclei, mitochondria, and cell debris were excluded as pellets by centrifugation at 10,000 × *g* for 5 min at 4 °C. The resulting supernatants were divided into the membrane-enriched fraction (pellet) and the cytoplasmic fraction (supernatant) by centrifugation at 100,000 × *g* for 1 h at 4 °C. Then, the membrane-enriched fraction (pellet) was washed with hypotonic buffer by passing through a 26-guage needle and re-centrifuged at 100,000 *g* for 45 min at 4 °C. Segregation of plasma membrane fraction was performed as described^22^. E-Cadherin (#3195, Cell Signaling) and N-Cadherin (610920, BD Biosciences) serves as a plasma membrane marker in western blotting.

### Quantitative Real-Time PCR

Total RNA was isolated with TRIZOL reagent (Invitrogen) according to the manufacturer’s protocol. Reverse transcription was performed with Moloney Murine Leukemia Virus Reverse Transcriptase (M-MLV RT) (Enzynomics). Transcript levels were determined by real-time PCR using a PowerUp^TM^ SYBR^TM^ Green Master Mix (Thermo Fisher Scientific) using the following synthetic oligonucleotides sets: human *APIP* #1 5′-GCGCAGGACAAGGAG CAT-3′ (forward), 5′-TTCTTCGAT GGCGAAGGTCC-3′ (reverse), human *APIP* #2 5′-GCGCAGGACAAGGAGCA-3′ (forward), 5′-CGATGGCGAAGGTCCACTTA-3′ (reverse); mouse *Apip* #1 5′-GCTCTCTCGCCTAATAGCGT-3′ (forward), 5′-TCTGGCTGAATGCGTTCCTT-3′ (reverse), mouse *Apip* #2 5′-TCTGGCTGTCAAGCTCAAGG-3′ (forward), 5′-CGCCTGAGGGAGCAATGTAG-3′ (reverse); rat *Apip* 5′-TAAGTGGGCCTCCAGCATCTA-3′ (forward), 5′-TCCTGTCCTGGAAACAGAAGG-3′ (reverse), rat *Gapdh* 5′-CAACTCCCTCAAGATTGTCAGCAA-3′ (forward), 5′-GGCATGGACTGTGGTCATGA-3′ (reverse); human *ADORA2B* 5′-ATGGAACCACGAATGAAAGC-3′ (forward), 5′-ATGTAGCTCATGGGGACCAC-3′ (reverse); mouse *Adora2b* 5′-TGCATGCCATCAACTGTATC-3′ (forward), 5′-TGGAAACTGTAGCGGAAGTC-3′ (reverse). Normalization of the gene of interest in samples was calculated using the ΔCt values as previously described^23^.

### Human Samples

All human tissue samples were obtained from patients under protocols approved by the Institutional Review Board of the Icahn School of Medicine at Mount Sinai. Gene expression was analyzed in human myocardium tissues obtained from patients with heart failure (*n* = 18) or from healthy donors (*n* = 29). Tissues were frozen in liquid nitrogen and stored at −80 °C.

### Statistics

All values are presented as mean ± standard error of the mean (SEM). Statistical analysis was performed using GraphPad Prism software (Version 5.01, GraphPad, La Jolla, CA). Statistical significance was determined by two-tailed *t* test (for groups of 2) or by one-way ANOVA (for groups of ≥ 3). Significant levels indicated are as follows: *P *<* 0.05, **P *<* 0.01, ***P *<* 0.001.

## Results

### Both APIP and ADORA2B are upregulated in the hearts of patients with heart failure

**Fig. 1:**
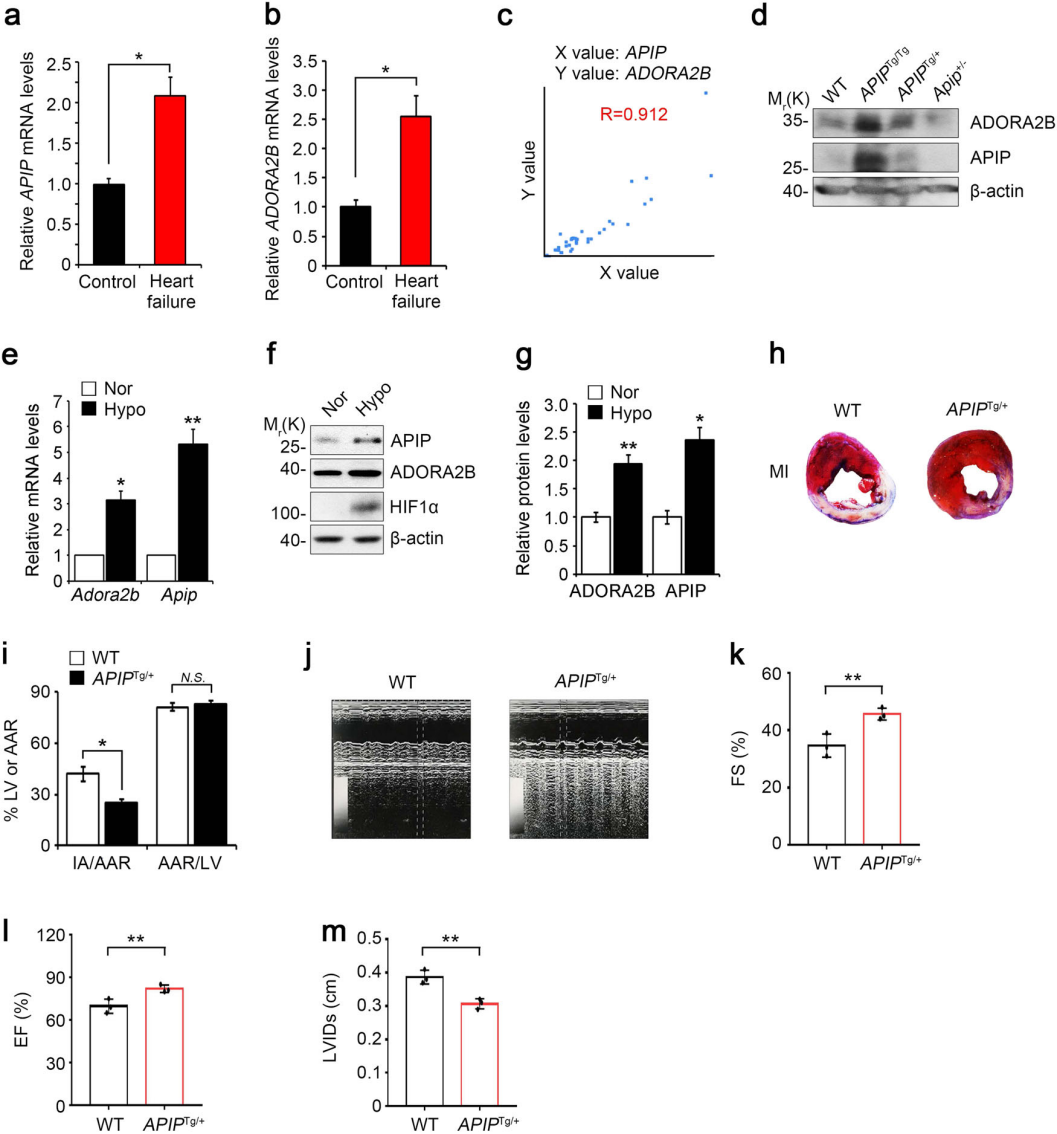
Cardiac overexpression of APIP found in patients with heart failure reduces infarct size and preserves cardiac function after myocardial infarction in mice. **a**–**c** Increase of both *Apip* and *Adora2b* transcripts in cardiac tissue from patients with heart failure. *Apip* (**a**) and *Adora2b* (**b**) transcripts in cardiac tissue from patients with heart failure or controls were quantified by real-time RT-PCR analysis (Control group: *n* = 29, Heart failure patient group: *n* = 18). Statistical significance was determined by Student’s *t* test. Levels of APIP and ADORA2B transcripts were plotted with mean Pearson correlation coefficient (0.912) (**c**). **d** APIP regulates ADORA2B in mice. Representative western blot showing ADORA2B protein levels in the heart of WT, *APIP*^Tg/Tg^, *APIP*^Tg/+^, and *Apip*^+/−^ mice at 4–5 months of age. **e**–**g** Increase of both *Apip* and *Adora2b* transcripts, and APIP protein in the hypoxic condition. Cultured primary cardiomyocytes were incubated with normoxic (Nor., 21% O_2_) or hypoxic (Hypo., 1% O_2_) condition for 6 h and total RNAs were analyzed by real-time RT-PCR analysis (**P < 0.01, *P < 0.05, two-tailed *t* test) (**e**). Cell lysates were analyzed by western blotting (**f**) and the signals on the blots were quantified by densitometric analysis (**P < 0.01, *P < 0.05, two-tailed *t* test) (**g**). **h**, **i** Reduction of infarct area in the heart of *APIP*^Tg/+^ mice. *APIP*^Tg/+^ mice or WT littermate were subjected to ligation of the left descending anterior coronary artery for 24 h and the hearts were then stained with Evans blue and 2,3,5-triphenyltetrazolium chloride (TTC). The Evans blue-perfused area, which is not at risk, was stained blue, viable myocardium was stained red, and infarcted myocardium appeared pale. The representative images are shown (**h**). AAR/LV indicates percentage of LV at risk, IA/AAR, infarcted area as percentage of risk area (Male, 12 weeks, *n* = 8 mice per group, *P *<* 0.05, *N.S*., not significant (*P* > 0.05), one-way ANOVA/Bonferroni) (**i**). **j**–**m** Restoration of cardiac function in *APIP*^Tg/+^ mice. *APIP*^Tg/+^ mice and WT littermates are subjected to ligation of the left descending anterior coronary artery. Left ventricular (LV) chamber dimensions and systolic function were measured at 3 weeks after the ligation surgery. Typical echocardiographic M-mode images are shown (**j**). Data are represented as the mean ± S.D. (male, 13–14 weeks, *n* = 3 mice per group, **P *<* 0.01, one-tailed *t* test). Parameters for cardiac function: FS fractional shortening, EF ejection fraction, LVIDs LV internal diameter at systole (**k**–**m**)

To understand the pathophysiological role of APIP in the heart under oxygen availability, we first investigated the expression levels of APIP in cardiac tissues prepared from 18 patients with ischemic heart disease (IHD), congestive heart failure (CHF), dilated cardiomyopathy (DCM), or heart failure (HF) and 29 healthy adults (Supplementary Table 1). Compared to that in the cardiac tissues of healthy controls, there was a significant increase in *APIP* mRNA levels in the diseased hearts (Fig. 1a). Consistent with the previous studies^24^, we found that the transcript levels of *ADORA2B*, a cytoprotective receptor^10–12^, were higher in the same heart samples of patients with heart failure than in controls (Fig. 1b). Interestingly, there was a correlation in the expression of *APIP* and *ADORA2B* mRNAs in the diseased hearts (mean Pearson correlation coefficient = 0.912) (Fig. 1c). Of interest, the levels of ADORA2B protein were influenced by APIP expression in the hearts of *APIP* transgenic (*APIP*^Tg/Tg^, *APIP*^Tg/+^) and *Apip* heterozygote knockout (*Apip*^+/−^) mice; the highest level of ADORA2B was detected in the hearts of *APIP*^Tg/Tg^ mice carrying double copies of human *APIP* transgene and the lowest level was observed in *Apip*^+/−^ mice (Fig. 1d).

### APIP ameliorates myocardial infarction and cardiac dysfunction in *APIP*^Tg/+^ mice

We then explored the expression of APIP in primary cardiomyocytes in response to hypoxia. Compared to the levels in normoxic conditions, the exposure of primary cardiomyocytes to hypoxic conditions (1% O_2_) resulted in a 5.3-fold increase in *APIP* mRNA levels (Fig. 1e) and a 2.4-fold increase in APIP protein levels (Fig. 1f, g). Under the same hypoxic conditions, we also found that Adora2b at the mRNA and protein levels increased 2- to 3-fold (Fig. 1e–g). It is interesting to note that APIP was induced with kinetics similar to those of HIF1α under hypoxia (Supplementary Fig. 1a).

The increase in APIP expression in the cardiomyocytes under hypoxia and in human patients with heart failure led us to investigate the role of APIP in MI. To address this, we generated APIP transgenic (*APIP*^Tg/+^) mice with ubiquitous expression of human APIP under the control of the β-actin-CMV promoter^17^. Compared to levels in age-matched wild-type (WT) littermates, the levels of APIP were approximately 3- to 5-fold higher in the hearts of *APIP*^Tg/+^ mice (Supplementary Fig. 1b, c). When *APIP*^Tg/+^ or WT mice were subjected to ligation of the left anterior descending coronary artery (LAD) for 24 h and the hearts were then stained with Evans blue and 2,3,5-triphenyltetrazolium chloride (TTC), we found that infarct sizes were 41.9 ± 4% at 24 h following the ligation in WT mice. On the other hand, the infarct sizes of *APIP*^Tg/+^ mice were significantly decreased under the same conditions (Fig. 1h, i).

To examine the effect of APIP on cardiac function after MI, we perform echocardiographic examinations at 3 weeks after LAD-ligation in *APIP*^Tg/+^ and WT mice. Analysis of echocardiographic M-mode images showed more restored cardiac function in *APIP*^Tg/+^ mice than WT littermates (Fig. 1j). Accordingly, a significant preservation of left ventricular fractional shortening and ejection fraction, and decrease in cardiac dilatation (LV internal diameter at systole, LVIDs) were seen in *APIP*^Tg/+^ mice compared with WT mice at 3 weeks after MI (Fig. 1k–m).

### APIP suppresses hypoxic cell death in cardiomyocytes via the AKT-HIF1α pathway

**Fig. 2:**
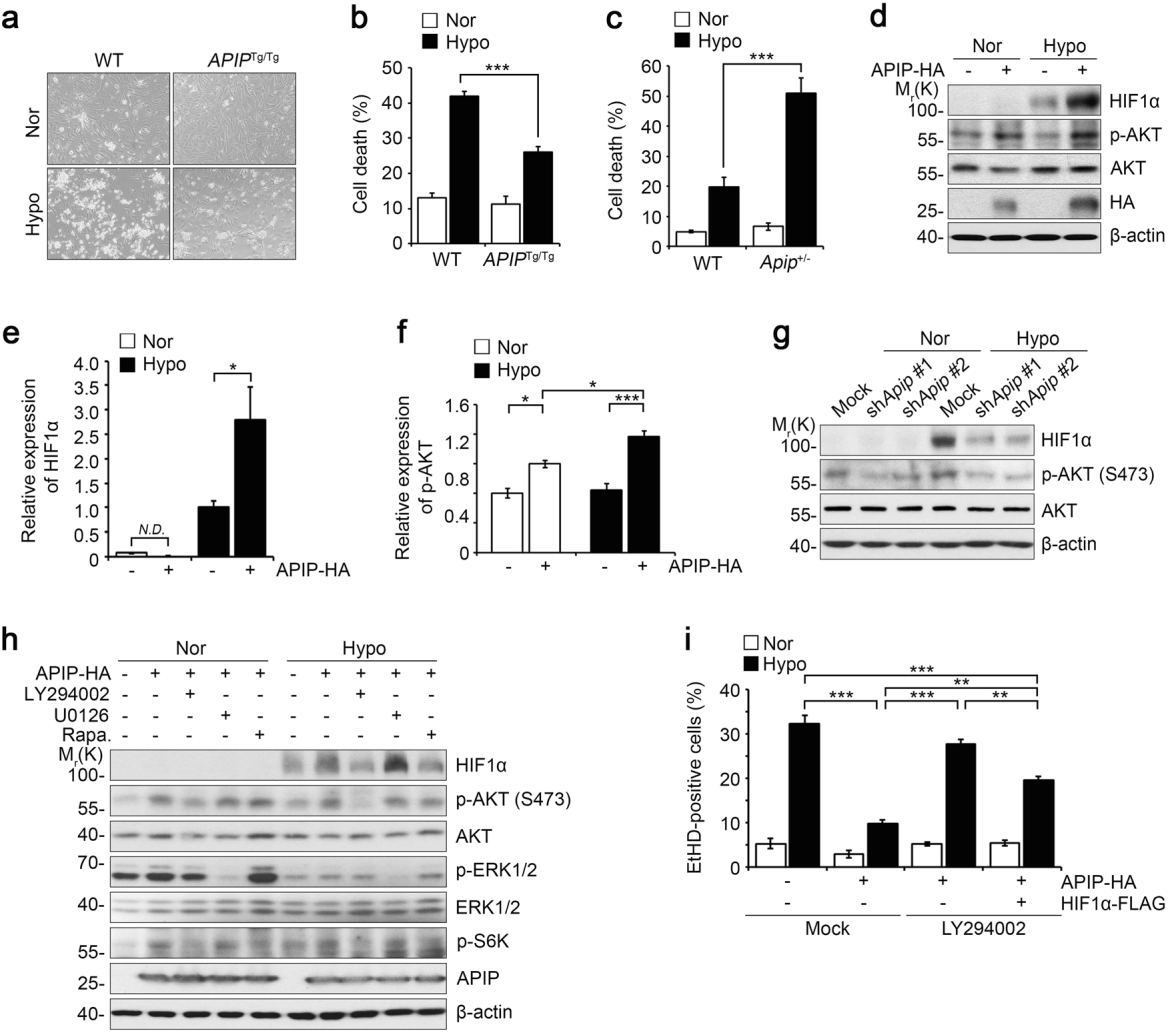
APIP suppresses cardiac cell death in vitro via the activation of AKT-HIF1α axis. **a**, **b** Suppression of cell death of primary cardiomyocytes in the hypoxic condition. Cardiomyocytes isolated from neonatal WT and *APIP*^Tg/Tg^ mice were exposed to normoxic (Nor) or hypoxic (Hypo) conditions for 36 h (**a**). The percentage of cell death was determined with the trypan blue exclusion assay (***P < 0.001, one-way ANOVA/Bonferroni, *n* = 3) (**b**). **c** Promoted cell death of *Apip*-haploinsufficient mouse (*Apip*^+/−^) cardiomyocytes under hypoxia. Primary cardiomyocytes were cultured from *Apip*^+/−^ mice and age-matched WT mice, exposed to hypoxic condition for 24 h, and dead cells were stained with propidium iodide (PI) (***P < 0.001, one-way ANOVA/Bonferroni). **d**–**f** Elevation of HIF1α and the phosphorylated AKT by APIP expression under hypoxia. H9c2 cells were transfected with pcDNA3-HA (Mock) or APIP-HA for 24 h, incubated under hypoxia for 6 h, and analyzed by western blotting (**d**). The signals of HIF1α and p-AKT on the blots were quantified by densitometric analysis (***P < 0.001, *P < 0.05, one-way ANOVA/Bonferroni). N.D. not determined (**e**, **f**). **g** Lack of HIF1α stabilization by *Apip* knockdown under hypoxia. H9c2 cells were transfected with pSUPER-neo (Mock) or *Apip* shRNAs (sh*Apip* #1 and sh*Apip* #2) for 24 h, incubated under hypoxia for 6 h, and analyzed by western blotting. **h** HIF1α stabilization by APIP depends on AKT and mTOR. H9c2 cells were transfected with pcDNA3-HA or APIP-HA for 24 h, treated with 30 μM LY294002, 10 μM U0126 or 1 μg/ml rapamycin for 2 h, and then incubated under hypoxia for 6 h. **i** Protection of hypoxic cell death by APIP-AKT requires HIF1α. H9c2 cells were transfected with pcDNA3-HA (Mock) or APIP-HA alone or together with HIF1α-FLAG for 24 h, exposed to hypoxic condition for 48 h in the presence or absence of 30 μM LY294002, and then stained with ethidium homodimer (EtHD) (***P < 0.001, **P < 0.01 one-way ANOVA/Bonferroni, *n* = 4)

To determine whether APIP directly influences cell death, we isolated cardiomyocytes from neonatal *APIP*^Tg/Tg^ and WT mice and incubated the cells under hypoxic conditions (1% O_2_). Under hypoxia, we observed 41.9 ± 1.3% cell death in WT cardiomyocytes. On the other hand, hypoxia-induced cell death was remarkably decreased in the cardiomyocytes of *APIP*^Tg/Tg^ mice, i.e., 25.9 ± 1.6% (Fig. 2a, b). We also evaluated the effect of APIP deficiency on the vulnerability of cardiomyocytes to hypoxic cell death. We found that cardiomyocytes cultured from *Apip*-haploinsufficient (*Apip*^+/−^) mice were more susceptible to hypoxic cell death than were WT cardiomyocytes (Fig. 2c). These results strongly suggest that APIP suppresses cardiomyocyte death under hypoxic stress.

We next examined the cellular mechanisms underlying the protective effects of APIP, using H9c2 cells which are frequently utilized as an in vitro model of acute MI under hypoxic conditions^25–29^. Interestingly, we found that the expression of HIF1α, a hypoxia-responsive transcription factor^30,31^, was increased by APIP overexpression in normoxic conditions and was more drastically induced under hypoxic conditions (Fig. 2d, e). There were no significant changes in the levels of *HIF1α* mRNA under the same conditions (data not shown), indicating that HIF1α accumulation is regulated mainly at the protein level. Parallel to the induction of HIF1α, the phosphorylation of AKT at Ser473 was significantly enhanced by APIP overexpression under hypoxia (Fig. 2d, f). Conversely, the knockdown of APIP expression reduced not only HIF1α levels but also AKT phosphorylation at Ser473 under hypoxia (Fig. 2g). We examined APIP shRNA efficiency in H9c2 cells with quantitative PCR analysis and found that APIP mRNA level was significantly reduced by sh*Apip* #1 or sh*Apip* #2 (Supplementary Fig. 2).

According to the previous reports^32,33^, both the PI3K-AKT and MAPK-ERK pathways play crucial roles in the post-translational regulation of HIF1α. We thus examined the effects of the pharmacological inhibitors of these kinases on the accumulation of HIF1α. As shown in Fig. 2h, HIF1α stabilization resulting from APIP overexpression and hypoxic stress was remarkably inhibited by the treatment with LY294002, a PI3K inhibitor, or rapamycin, an mTOR inhibitor, suggesting that PI3K-AKT and mTOR contribute to the increase in HIF1α levels via APIP under hypoxia. The blockade of ERK did not affect the protein level of HIF1α. In addition, we investigated the significance of the PI3K-AKT axis in hypoxia-induced cell death. Similar to the results observed in cardiomyocytes, hypoxia-induced cell death was inhibited by APIP overexpression in H9c2 cells (Fig. 2i). The inhibition of hypoxia-induced cell death by APIP significantly disappeared upon LY294002 treatment. Further, under the same set of conditions, this inhibition of hypoxia-induced cell death by APIP was partially restored by the ectopic expression of HIF1α, even in the presence of LY294002 (Fig. 2i). These data suggest that APIP inhibits hypoxic cell death via the AKT-HIF1α axis.

### APIP interacts with ADORA2B for cardioprotection against hypoxic injury

**Fig. 3:**
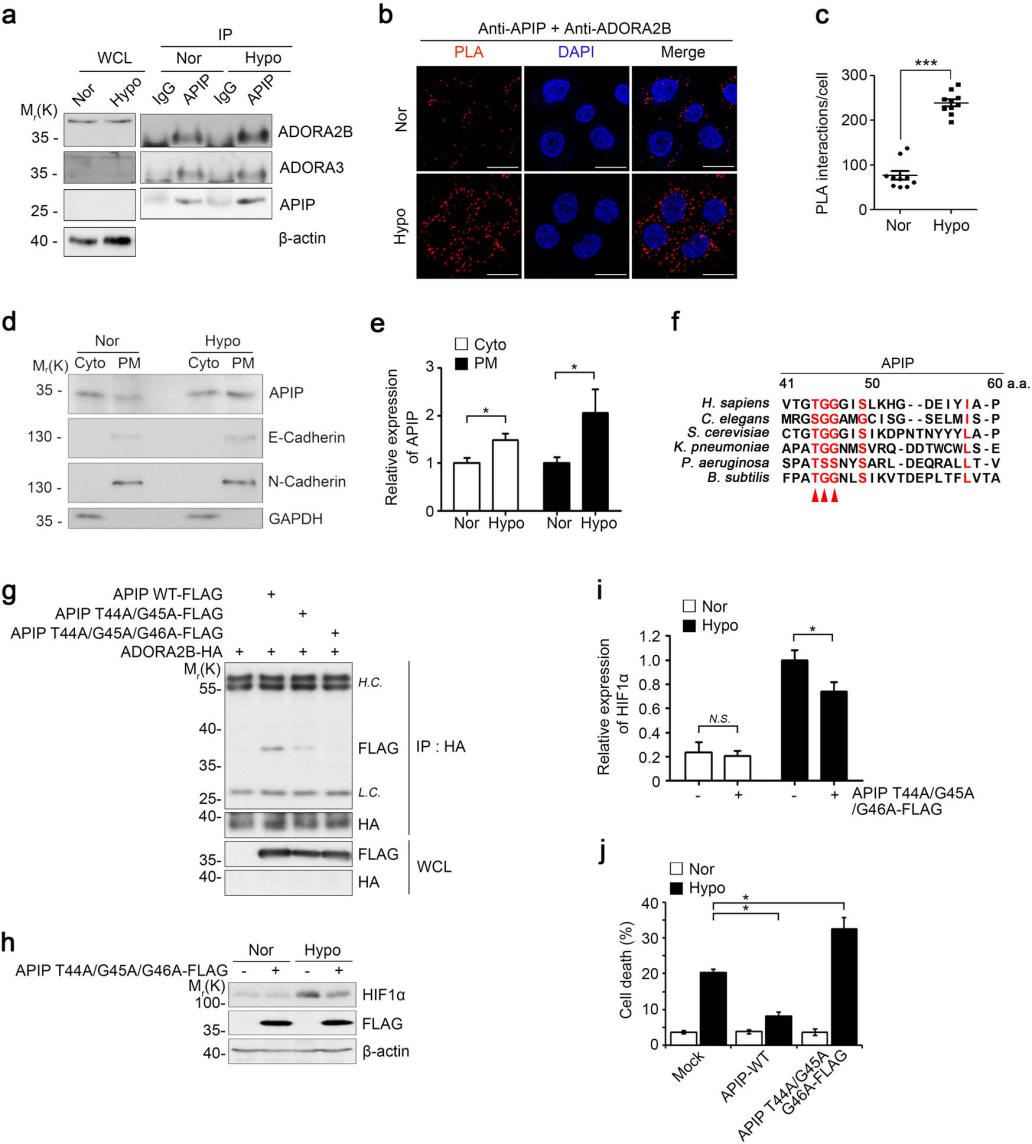
APIP interacts with ADORA2B to inhibit cell death under hypoxia. **a** APIP binds to ADORA2B under hypoxia. Primary cardiomyocytes were incubated in normoxic (Nor) or hypoxic (Hypo) condition for 6 h and then analyzed by immunoprecipitation (IP) assays. **b**, **c** In situ PLA detection of APIP-ADORA2B interaction under hypoxia. HeLa cells were incubated under the normoxic (Nor) and hypoxic (Hypo) condition for 6 h. Scale bars, 20 µm (**b**). Values represent the numbers of PLA dots per cell (**c**). Values represent the mean ± SD (*n* = 10 cells per each group). **d**, **e** Recruitment of APIP to the plasma membrane under hypoxia. HEK293T cells were incubated in normoxic (Nor) or hypoxic (Hypo) condition for 6 h and cell extracts were fractionated into the cytoplasmic (Cyto) and plasma membrane (PM) fractions. The fractions were analyzed with western blotting (**d**) and signals on the blots were quantified by densitometric analysis (*n* = 4, *P < 0.05, one-tailed *t* test) (**e**). APIP expression levels were normalized by those of GAPDH (Cyto) or N-Cadherin (PM). **f** Alignment of the N-terminal region (residues 41st–60th) of APIP among different species. The sequences are retrieved from UniProtKB database (http://www.uniprot.org). Arrowheads indicate conserved residues among the species. **g** Involvement of the N-terminal _44_Thr-Gly-Gly_46_ motif of APIP in the binding to ADORA2B. HEK293T cells were transfected with ADORA2B-HA and FLAG-APIP, FLAG-APIP T44A/G45A, or FLAG-APIP T44A/G45A/G46A for 24 h and analyzed by immunoprecipitation (IP) assays using HA antibody. **h**, **i** APIP T44A/G45A/G46A mutant does not stabilize HIF1α protein under hypoxia. H9c2 cells were transfected with p3XFLAG (Mock) or FLAG-APIP T44A/G45A/G46A for 24 h and incubated under normoxic (Nor) or hypoxic (Hypo) condition for 6 h. Cell lysates were analyzed by western blotting (**h**) and the signals of HIF1α on the blot were quantified by densitometric analysis (*n* = 5, *P < 0.05, *N.S*., Not significant, one-tailed *t* test) (**i**). **j** APIP T44A/G45A/G46A mutant fails to suppress hypoxic cell death. H9c2 cells transfected with p3XFLAG (Mock), FLAG-APIP, or FLAG-APIP T44A/G45A/G46A were exposed to hypoxic condition for 24 h (*P < 0.05, one-way ANOVA/Bonferroni)

Given that adenosine receptor signaling is important for myocardial adaptation to ischemia or hypoxia^12,34,35^, we hypothesized that this pathway is involved in the cardioprotective function of APIP. To evaluate this hypothesis, we examined whether APIP interacts with adenosine receptors. An immunoprecipitation assay revealed that among ADORA subtypes, APIP-FLAG interacted with ADORA2B-HA and ADORA3-HA, but not with ADORA1-HA and ADORA2A-HA in the transfected cells (Supplementary Fig. 4a). Since ADORA2B is the main receptor promoting survival in cardiomyocytes under hypoxic stress^12,36^, we examined the interaction between APIP and ADORA2B under hypoxia in cardiomyocytes. With an immunoprecipitation analysis, we confirmed that APIP interacted with ADORA2B, and this interaction increased under hypoxia, while the interaction between APIP and ADORA3 was not changed (Fig. 3a), suggesting that APIP interacts with different adenosine receptors depending on oxygen availability. In addition, the results of an *in situ* proximity-ligation assay provided further evidence for the interaction of endogenous APIP with ADORA2B and its substantial enhancement under hypoxia (Fig. 3b, c, Supplementary Fig. 3). Moreover, we performed cell fractionation experiments and found the consistent results showing enhanced recruitment of APIP into the plasma membrane under hypoxia (Fig. 3d, e), while APIP in normoxic cells is found mainly in the cytosol^37–39^. Thus, APIP interacts with ADORA2B in cardiomyocytes, upon hypoxic stress.

To identify the region of APIP that interacts with ADORA2B, we generated various APIP deletion mutants and examined their interactions with ADORA2B. Immunoprecipitation assays revealed that unlike other APIP N-terminal deletion (ΔN) mutants, the APIP ΔN50 and ΔN60 mutants lacking 50 and 60 amino acids, respectively, in the N-terminus did not bind to ADORA2B (Supplementary Fig. 4b–d), suggesting that the N-terminal region between the 41^st^ and 60^th^ amino acids of APIP is required for the interaction with ADOR2B. An amino acid sequence comparison of this region revealed that the Thr44, Gly45, and Gly46 residues are highly conserved among different species (Fig. 3f). We then generated additional APIP mutants by replacing these residues with alanine. Notably, the APIP T44A/G45A or APIP T44A/G45A/G46A mutant harboring double or triple point mutations at these residues failed to interact with ADORA2B (Fig. 3g). With reverse IP assays, we confirmed that the N-terminal _44_Thr-Gly-Gly_46_ motif of APIP was essential for its interaction with ADORA2B (Supplementary Fig. 4e). Moreover, under hypoxia, the APIP T44A/G45A/G46A mutant did not increase HIF1α protein level so much as WT APIP (Fig. 3h, i) or failed to inhibit cell death (Fig. 3j). This mutant rather exhibited the opposite activity in cell death to that of WT APIP. Thus, the protective effect of APIP against hypoxic damage is accomplished by the interaction of APIP with ADORA2B.

### ADORA2B-CREB-HIF1α signaling under hypoxia depends on APIP

Next, we attempted to demonstrate the functional association of APIP with ADORA2B signaling or *vice versa*. We conducted some experiments in HeLa cells and confirmed the preservation of APIP function in HeLa cells under hypoxia. Under both normoxic and hypoxic conditions, we found that the levels of APIP were decreased by ADORA2B knockdown (Supplementary Fig. 5a). Conversely, treatment with BAY60-6583, a selective agonist for ADORA2B with a cardioprotective function against MI^36,40^, increased APIP levels in a dose-dependent manner (Supplementary Fig. 5b). To validate the role of APIP in ADORA2B-mediated cytoprotection under hypoxia, we analyzed HIF1α levels and hypoxic cell death in APIP knockdown cells. We observed the overexpression of ADORA2B greatly induced HIF1α levels and suppressed cell death under hypoxic conditions, but this effect of ADORA2B was also blocked by silencing APIP expression (Supplementary Fig. 5c, d). It is interesting to note that the level of APIP was also increased by the overexpression of ADORA2B. These results indicate that APIP is essential for ADORA2B-mediated accumulation of HIF1α and for blocking hypoxic cell death.

**Fig. 4:**
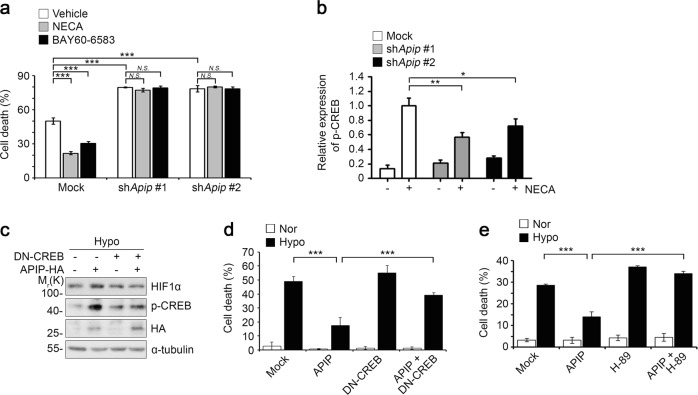
The CREB-HIF1α signaling triggered by ADORA2B depends on APIP. **a** Essential role of APIP in ADORA2B-mediated cytoprotection against hypoxia. H9c2 cells were transfected with pSUPER-neo (Mock) or *Apip* shRNAs (sh*Apip* #1 or sh*Apip* #2) for 24 h and incubated with serum-free medium for 24 h. Cells were then treated with 10 μM NECA or 10 μM BAY 60-6583 for 10 min and incubated under hypoxic condition for 24 h (***P *<* 0.001, *N.S*., not significant (*P* > 0.05), one-way ANOVA/Bonferroni). **b** Role of APIP in ADORA2B-mediated CREB activation under hypoxia. H9c2 cells were transfected with pSUPER-neo (Mock) or *Apip* shRNAs (sh*Apip* #1 or sh*Apip* #2) for 24 h and incubated with serum-free medium for 24 h. Cells were then left untreated or treated with 10 μM NECA for 10 min and incubated under the hypoxic condition for 2 h. Cell lysates were analyzed by western blotting and the signals of p-CREB were quantified by densitometric analysis (*n* = 4, **P < 0.01, *P < 0.05, one-tailed *t* test). **c**, **d** APIP regulates HIF1α stabilization and cell death through CREB under hypoxia. H9c2 cells were transfected with dominant negative CREB (DN-CREB) and APIP-HA for 24 h and incubated under the hypoxic condition for 3 h (**c**) and 24 h (**d**). Cell lysates were analyzed by western blotting (**c**) and cell death rates were determined (***P < 0.001, one-way ANOVA/Bonferroni, *n* = 4) (**d**). **e** APIP protects cells against hypoxia through PKA. H9c2 cells were transfected with pEGFP-N1 and either pcDNA3-HA (Mock) or APIP-HA, and then incubated with serum-free medium for 24 h. After pre-treatment with vehicle (Mock) or 10 μM H-89 for 5 min, cells were incubated under normoxic (Nor) or hypoxic (Hypo) condition for 18 h (***P < 0.001, one-way ANOVA/Bonferroni, *n* = 3)

Treatment with 5′-*N*-ethylcarboxamidoadenosine (NECA), a non-selective adenosine receptor agonist, or BAY60-6583 suppressed hypoxic cell death in H9c2 cells (Fig. 4a). However, the anti-cell death effects of NECA or BAY60-6583 were abolished by APIP knockdown. As a downstream event in ADORA2B activation, the PKA-CREB signaling pathway plays a role in cardioprotection against MI^12^. When we examined the levels of CREB activation in APIP knockdown cells, we observed that the elevation in phosphorylated CREB at Ser133 by NECA was inhibited by APIP knockdown (Fig. 4b). In addition, we examined the role of CREB in APIP-mediated cardioprotection and found that the expression of a dominant-negative mutant (DN) of CREB^41^ impaired the APIP-mediated accumulation of HIF1α (Fig. 4c) and APIP-mediated protection of hypoxic cell death (Fig. 4d). A similar effect with DN-CREB was observed when cells were treated with H-89, an inhibitor of PKA that phosphorylates CREB; treatment with H-89 also counteracted APIP function (Fig. 4e). To sum up, the cardioprotective function of ADORA2B via a PKA-CREB signaling pathway is regulated by APIP.

### Reduced potency of the ADORA2B D296G SNP in cardioprotection and interactions with APIP under hypoxia

**Fig. 5:**
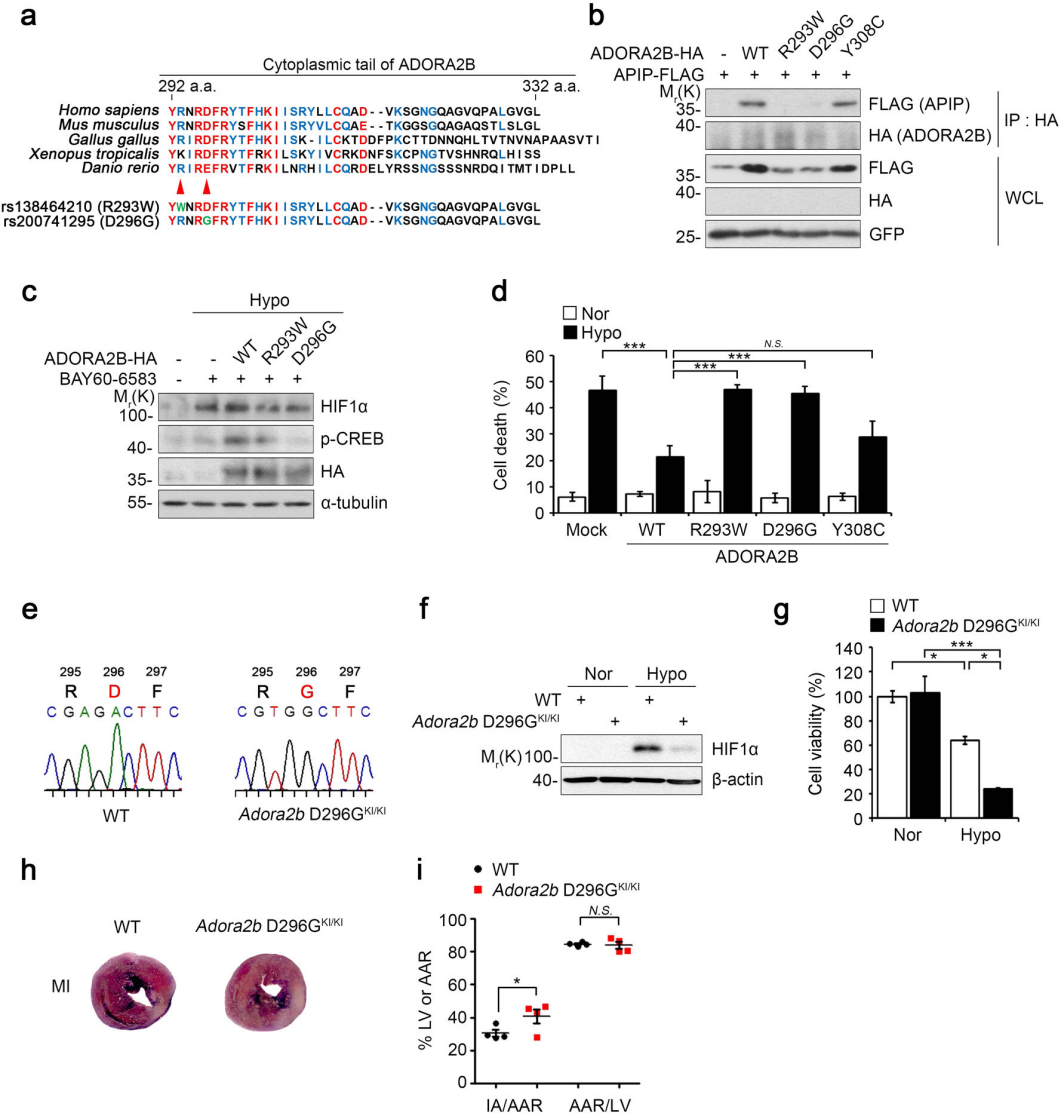
ADORA2B SNP D296G do not protect cardiomyocytes under hypoxia and the heart from MI in *Adora2b* D296G^KI/KI^ mice. **a** Comparison of ADORA2B C-terminal amino acid sequences among different species. **b** Arg293 and Asp296 of ADORA2B are important to interact with APIP. HEK293T cells were transfected with FLAG-APIP and pDEST-HA (-), ADORA2B-HA, ADORA2B R293W-HA, ADORA2B D296G-HA, or ADORA2B Y308C-HA for 24 h and then analyzed by immunoprecipitation (IP) assay. **c** ADORA2B R293W and D296G mutants do not induce CREB phosphorylation and HIF1α accumulation under hypoxia. H9c2 cells were transfected with ADORA2B-HA, ADORA2B R293W-HA, or ADORA2B D296G-HA for 24 h, pre-treated with 10 μM BAY60-6583 for 10 min, and then incubated under hypoxic condition for 2.5 h. **d** ADORA2B R293W and D296G mutants fail to suppress hypoxia-induced cell death. H9c2 cells were transfected with EGFP and ADORA2B-HA, ADORA2B R293W-HA, or ADORA2B D296G-HA for 24 h, incubated under hypoxia for 24 h, and cell death rates were measured (***P *<* 0.001, *N.S*., not significant (*P* > 0.05), one-way ANOVA/Bonferroni). **e** Generation of *Adora2b* D296G knock-in mice. Direct sequencing analysis of the PCR product covering the mutation site in wild-type (WT) and homozygous *Adora2b* D296G knock-in mice (*Adora2b* D296G^KI/KI^). **f**, **g** Cultured cardiomyocytes from *Adora2b* D296G knock-in mice do not stabilize HIF1α and are vulnerable to cell death under hypoxic conditions. Cardiomyocytes isolated from neonatal WT and *Adora2b* D296G knock-in mice were exposed to normoxic (Nor) or hypoxic (Hypo) condition for 6 h (**f**) or 24 h (**g**). Cell lysates were examined by western blot analysis (**f**). Cell viability was determined by using EZ-CYTOX^TM^ cell viability kit (***P < 0.001, *P < 0.05, one-way ANOVA/Bonferroni) (**g**). **h**, **i** Increase of infarct area in the heart of *Adora2b* D296G^KI/KI^ mice. *Adora2b* D296G^KI/KI^ mice or WT littermate were subjected to ligation of the left descending anterior coronary artery for 18 h and the hearts were then stained with Evans blue and 2,3,5-triphenyltetrazolium chloride (TTC) as in Fig. 1h. The representative images are shown (**h**). AAR/LV indicates percentage of LV at risk, IA/AAR, infarcted area as percentage of risk area (Male, 12 weeks, *n* = 4 mice per group, *P *<* 0.05, *N.S*., not significant (*P* > 0.05), one-way ANOVA/Dunnett) (**i**)

Notably, we found that Arg293 and Asp296 residues of ADORA2B have polymorphisms, rs138464210 and rs200741295 (Fig. 5a). Considering amino acid sequence homology in the cytoplasmic tail of ADORA2B among various species, we found that these Arg293 and Asp296 residues are highly conserved. We thus determined the relevance of these residues for the interaction between ADORA2B and APIP. Immunoprecipitation assays revealed that the ADORA2B R293W mutant in which Arg293 is replaced with Trp and the ADORA2B D296G mutant in which Asp296 is replaced with Gly did not bind to APIP, suggesting that the ADORA2B Arg293 and Asp296 residues are critical for the interaction with APIP (Fig. 5b). While the Tyr308 residue of ADORA2B is also highly conserved, the ADORA2B Y308C mutant in which Tyr308 is replaced with Cys bound to APIP. We also found that unlike WT ADORA2B, the ADORA2B R293W and D296G mutants did not induce HIF1α accumulation or CREB activation under hypoxic stress (Fig. 5c, Supplementary Fig. 6). Furthermore, ADORA2B R293W and D296G mutations did not suppress hypoxic cell death (Fig. 5d). These results indicate that these SNPs in the Arg293 and Asp296 residues of ADORA2B cause a loss of binding to APIP and ADORA2B signaling for the protection of cardiac cell death via the CREB-HIF1α pathway.

To elucidate the in vivo and pathological importance of ADORA2B SNPs, we generated *Adora2b* D296G knock-in (KI) mice (*Adora2b* D296G^KI/+^) using CRISPR/Cas9-derived RNA-guided endonucleases (RGEN) (Fig. 5e, Supplementary Fig. 7a–c). We crossed the *Adora2b* D296G^KI/+^ mice with WT mice and generated homozygous *Adora2b* D296G KI mice (*Adora2b* D296G^KI/KI^) by crossing the descendent *Adora2b* D296G^KI/+^ mice. When we analyzed primary cardiomyocytes cultured from *Adora2b* D296G^KI/KI^ mice, we found that the accumulation of HIF1α, which was observed in WT cardiomyocytes under hypoxia, was markedly reduced in primary cardiomyocytes of *Adora2b* D296G^KI/KI^ mice (Fig. 5f). In addition, *Adora2b* D296G^KI/KI^ cardiomyocytes were more sensitive to hypoxic damage than WT cardiomyocytes (Fig. 5g). Moreover, myocardial infarct areas in the hearts of *Adora2b* D296G^KI/KI^ mice were significantly greater than those of age-matched WT mice (Fig. 5h, i). Together, these results indicate that the ADORA2B D296G SNP, which resulted in the failure to bind to APIP, displays impaired protection against myocardial damage under hypoxic conditions.

## Discussion

On the basis of our analysis of cardiac function in *APIP*^Tg/+^ and *APIP*^+/−^ mice, we found that APIP plays a crucial role in the protection of cardiomyocytes from cell death and preservation of cardiac function under MI. Interestingly, we observed similar expression patterns of APIP and ADORA2B in the hearts of patients with heart failure and of *APIP*^Tg/+^ and *APIP*^+/^^−^ mice. Given that the increased expression of APIP regulates ADORA2B level and protects the heart from MI, the increase of APIP as well as ADORA2B in the hearts of patients with heart failure is likely associated with signaling of cardioprotection during heart failure. In addition, we believe that APIP might be a critical regulator of ischemic preconditioning in myocardial protection, which is an interesting issue to be pursued further in the future. More, our observation that APIP regulates the AKT-HIF1α pathway and potentiates ADORA2B-PKA-CREB signaling raises a possibility that APIP might attenuate MI by driving the utilization of the oxygen-efficient carbohydrate-dependent metabolic pathway^12,42^.

Comparison of the amino acid sequence revealed that APIP has aldolase domain. Unlike the prediction, APIP was recently shown to have MtnB enzymatic activity in the methionine salvage pathway^14,37^. However, we found that the cardioprotective function of APIP is not associated with MtnB enzymatic activity because APIP C97A, H115A, and E139A mutants lacking this activity have similar effects as those of WT APIP on HIF1α signaling under hypoxia (data not shown). Rather, the ability of APIP to inhibit pyroptosis was proposed to be associated with MtnB activity^15^. While it is still not clear how APIP regulates these two distinct activities, the interaction of APIP with ADORA2B is important for the regulation of ADORA2B-PKA-CREB signaling in the cardioprotection. Meanwhile, we have generated *Apip* E139A knock-in mouse which has a mutation in the catalytic site of APIP and generated conditional *APIP* knockout mice using Cre-loxP system, and are waiting for the analysis of these mice to clearly answer that question.

Adenosine-specific receptors are divided into four subtypes—ADORA1, ADORA2A, ADORA2B, and ADORA3—based on their ability to either inhibit (via ADORA1 or ADORA3) or stimulate (via ADORA2A or ADORA2B) adenylate cyclase activity in response to adenosine binding^43,44^. Interestingly, our results showed that APIP binds to ADORA2B and ADORA3 among the four adenosine receptor subtypes in culture. While its binding to ADORA2B was significantly increased under hypoxic conditions, binding to ADORA3 was rather not changed. We believe that this interaction between APIP and ADORA2B under hypoxia leads to the stabilization of both proteins, resulting in stimulation of adenylate cyclase to increase intracellular cAMP levels. While the mechanism by which APIP stabilizes ADORA2B is not clear yet, we have an evidence that APIP regulates the degradation of ADORA2B protein through modulating the lysosomal pathway; the decrease in ADORA2B levels in APIP knockdown cells was rescued by the inhibition of lysosomes (Supplementary Fig. 8a–c). Given that cell surface proteins are degraded within lysosomes by endocytosis^45^, ADORA2B endocytosis for lysosomal degradation might be inhibited by APIP.

Until now, genetic factors underlying the predisposition to MI and involved in the regulation of the pathogenesis of MI have been largely unknown. ADORA2B D296G is the first SNP (rs200741295) found in human hearts (NCBI dbSNP database, http://www.ncbi.nlm.nih.gov/SNP/) and results in a missense mutation in ADORA2B. Interestingly, the ADORA2B D296G mutant failed to bind to APIP in primary cardiomyocytes and potentiated MI, as seen in *Adora2b* D296G^KI/KI^ mice. To evaluate the frequency and pathological relevance of this mutation to MI in human hearts, we performed a small-scale DNA sequencing analysis of heart samples biopsied from 356 patients with MI and 100 patients with non-MI but did not detect this mutation. Thus, the frequency of this mutation is not high but further studies are needed with larger numbers of patients with MI.

**Fig. 6:**
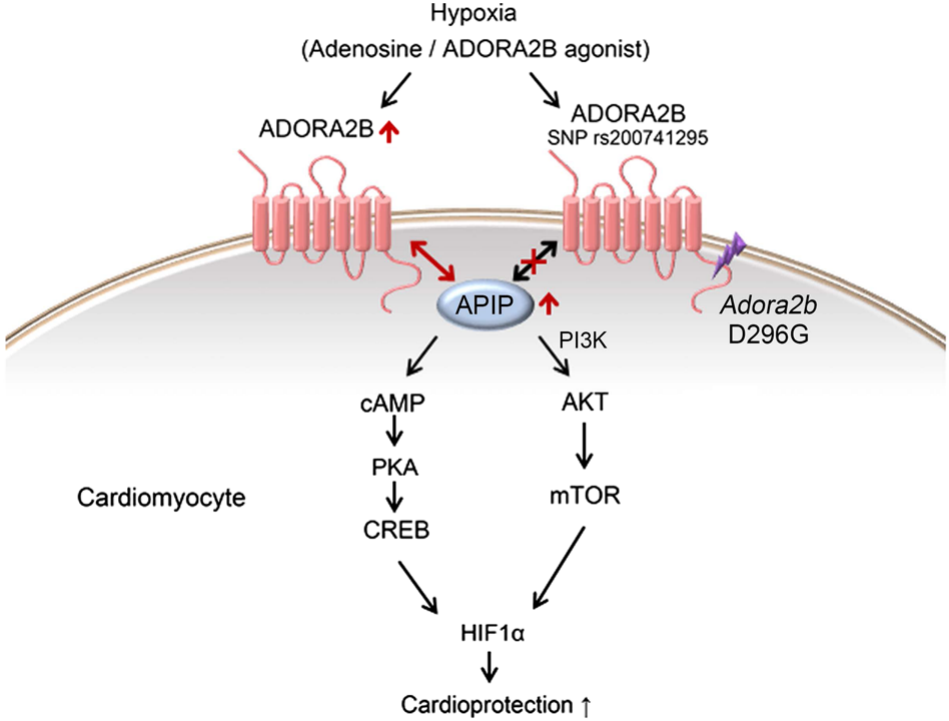
A mechanistic model showing the cardioprotective role of APIP in MI. APIP is increased to protect the heart from MI by increasing and binding to ADORA2B to generate survival signals through the PKA-CREB and AKT-mTOR pathways, leading to the accumulation of HIF1α. In contrast, ADORA2B SNP D296G cannot bind to APIP and thus fails to generate the signals

Considering therapeutic potential, the ADORA2B D296G SNP or low level of ADORA2B in the heart of patients with heart failure would not respond well to the therapy employing an ADORA2B agonist. On the contrary, when we intentionally increased APIP level by overexpression in cardiomyocytes harboring low levels of ADORA2B or D296G SNP, we found that the cardioprotective effect of the ADORA2B agonist was greatly enhanced. Thus, this report provides a new opportunity for the use of APIP in the ADORA2B therapeutics to increase the efficacy or overcome the defective protection of ADORA2B SNP. Of course, APIP itself can also be considered to be employed as a direct therapeutic approach to reduce cardiac damages or function during MI. In conclusion, our results showed that APIP is upregulated in cardiomyocytes exposed to hypoxia to potentiate ADORA2B signaling and thus protect cardiomyocytes from ischemic injury (Fig. 6). The ADORA2B D296G SNP might worsen MI in patients as a result of the protein’s lack of interaction with APIP. Thus, our findings reveal a critical role of APIP in the infarct damage of hearts under MI.

## Supplementary information

Supplementary Figure 1

Supplementary Figure 2

Supplementary Figure 3

Supplementary Figure 4

Supplementary Figure 5

Supplementary Figure 6

Supplementary Figure 7

Supplementary Figure 8

Supplementary Table 1

Supplementary figure legends
