# Supplementary figures and images for: Cardioprotective role of APIP in myocardial infarction through ADORA2B
Source: Cell Death Dis. 2019 Jul 1;10(7):511. doi: 10.1038/s41419-019-1746-3 (PMC6602929; doi:10.1038/s41419-019-1746-3)

Supplementary Figure 1.

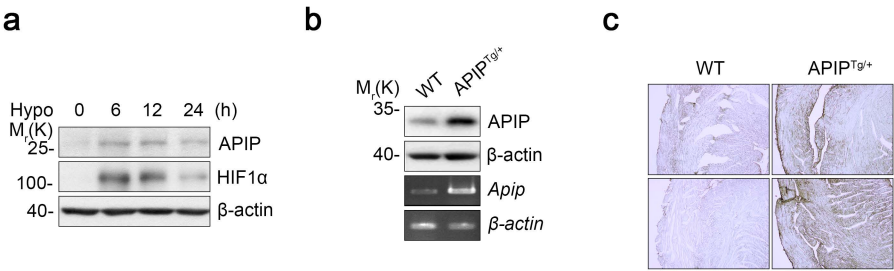

Supplement: Supplementary file 1 — Supplementary Figure 1 [file 41419_2019_1746_MOESM1_ESM.pdf]

Supplementary Figure 2.

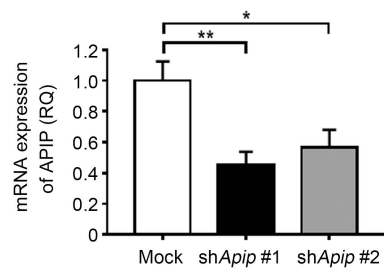

Supplement: Supplementary file 2 — Supplementary Figure 2 [file 41419_2019_1746_MOESM2_ESM.pdf]

Supplementary Figure 3.

**a**

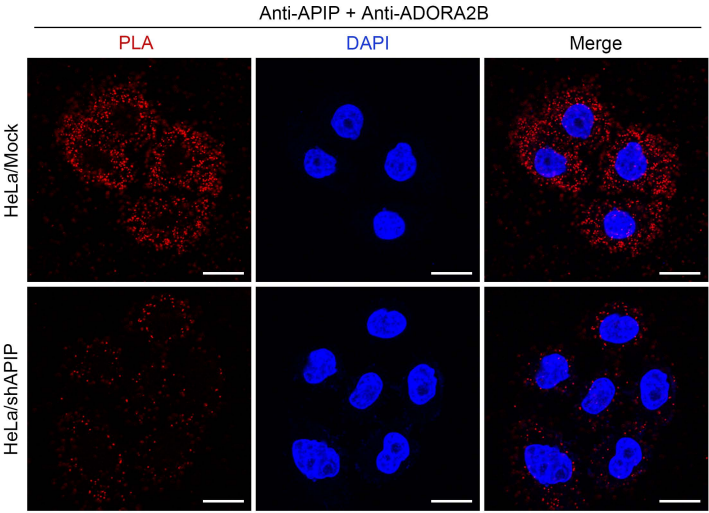

**b**

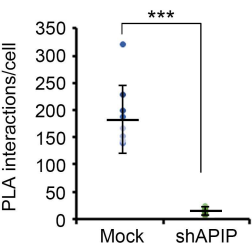

Supplement: Supplementary file 3 — Supplementary Figure 3 [file 41419_2019_1746_MOESM3_ESM.pdf]

Supplementary Figure 4.

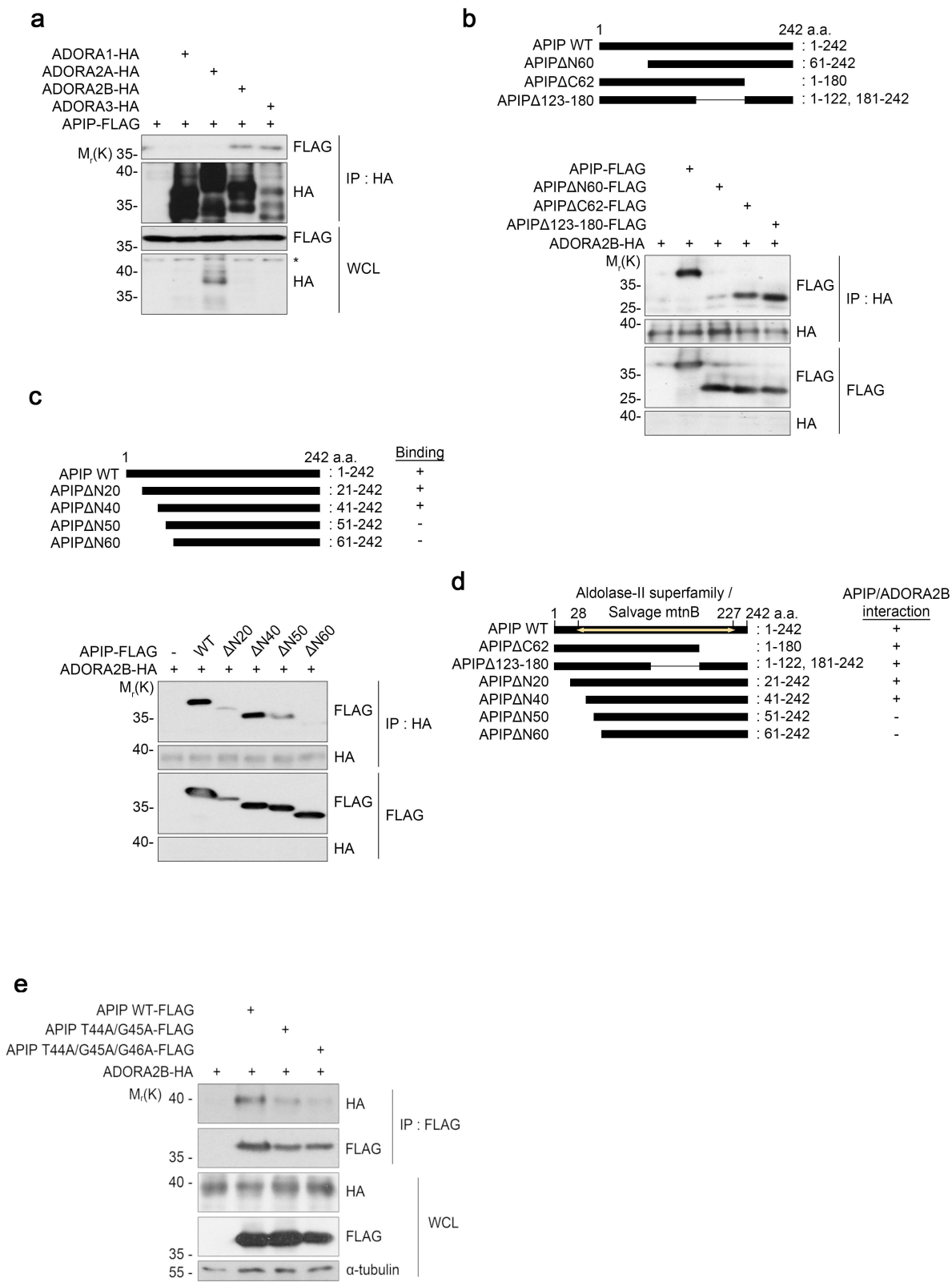

Supplement: Supplementary file 4 — Supplementary Figure 4 [file 41419_2019_1746_MOESM4_ESM.pdf]

Supplementary Figure 5.

**a**

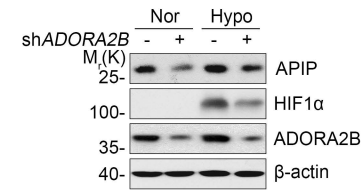

**b**

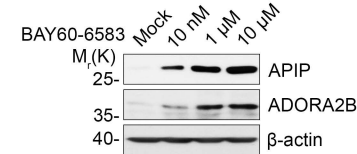

**c**

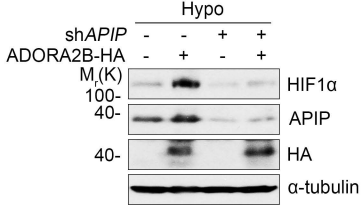

**d**

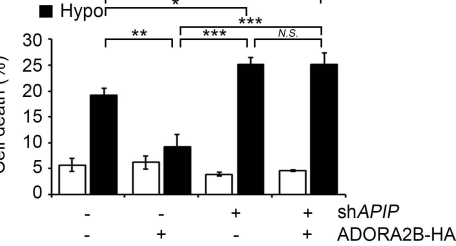

Supplement: Supplementary file 5 — Supplementary Figure 5 [file 41419_2019_1746_MOESM5_ESM.pdf]

Supplementary Figure 6.

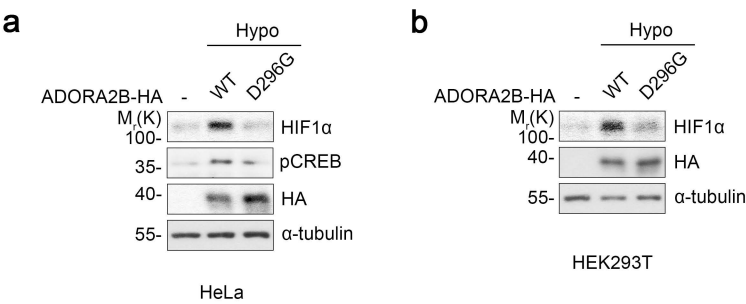

Supplement: Supplementary file 6 — Supplementary Figure 6 [file 41419_2019_1746_MOESM6_ESM.pdf]

Supplementary Figure 8.

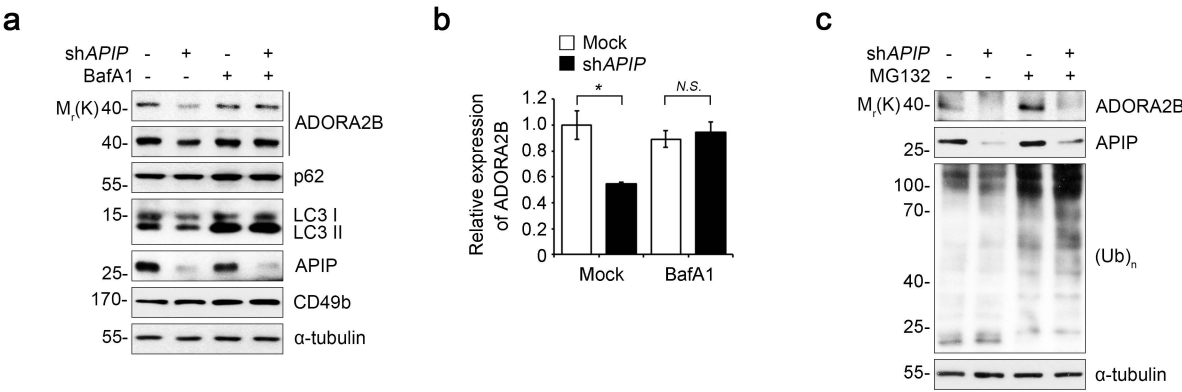

Supplement: Supplementary file 8 — Supplementary Figure 8 [file 41419_2019_1746_MOESM8_ESM.pdf]
