# Supplementary material for: Cardioprotective role of APIP in myocardial infarction through ADORA2B
Source: Cell Death Dis. 2019 Jul 1;10(7):511. doi: 10.1038/s41419-019-1746-3 (PMC6602929; doi:10.1038/s41419-019-1746-3)

Supplementary Figure 7.

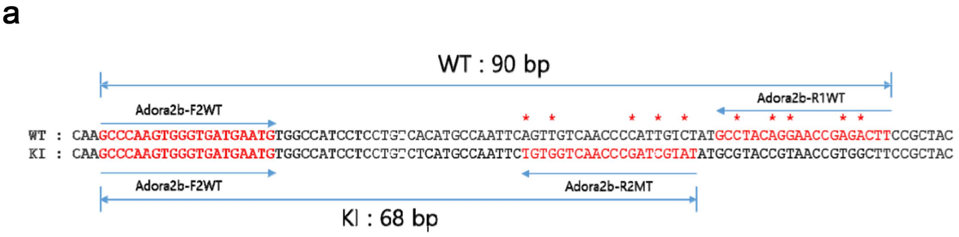

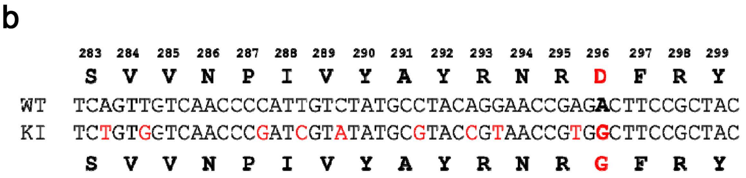

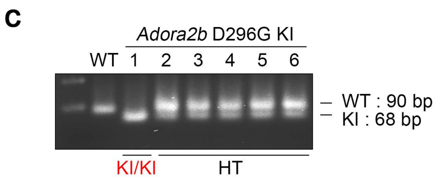

Supplement: Supplementary file 7 — Supplementary Figure 7 [file 41419_2019_1746_MOESM7_ESM.pdf]
