# Supplementary material for: Cardioprotective role of APIP in myocardial infarction through ADORA2B
Source: Cell Death Dis. 2019 Jul 1;10(7):511. doi: 10.1038/s41419-019-1746-3 (PMC6602929; doi:10.1038/s41419-019-1746-3)
Supplement: Supplementary file 9 — Supplementary Table 1 [file 41419_2019_1746_MOESM9_ESM.pdf]

Supplementary Table 1.

Control					Heart failure				
	ANONYMIZATION CODE	DISEASE	AGE	SEX		ANONYMIZATION CODE	DISEASE	AGE	SEX
N1	Donor 1	Donor/Control	52	F	F1	HF 1	IHD	48	M
N2	Donor 2	Control	?	?	F2	HF 2	IDC	61	M
N3	Donor 3	Normal	58	F	F3	HF 3	CHF	66	F
N4	Donor 4	Control	58	F	F4	HF 4	DCM		F
N5	Donor 5	Control	54	M	F5	HF 5	DCM	66	F
N6	Donor 6	Control	51	M	F6	HF 6	IHD	69	F
N7	Donor 7	Donor	61	F	F7	HF7	HF	?	?
N8	Donor 8	Donor	61	F	F8	NDRI 3	HF	61	M
N9	NDRI 4	normal	50	F	F9	NDRI 2	HF	48	M
N10	NDRI 8	normal	61	M	F10	NDRI 7	HF	71	M
N11	NDRI 9	normal	58	M	F11	106	DCM	65	CF
N12	13	NORMAL	17	M	F12	123	DCM	47	M
N13	29	NORMAL	24	F	F13	143	DCM	55	CF
N14	44	NORMAL	33	CM	F14	145	DCM	57	CM
N15	48	NORMAL	69	F	F15	152	DCM	25	HM
N16	54	NORMAL	35	CM	F17	197	DCM	28	CM
N17	55	NORMAL	16	F	F18	218	DCM	60	CM
N18	71	NORMAL	71	CF	F19	234	DCM	36	M
N19	75	NORMAL	58	CM					
N20	76	NORMAL	61	CF					
N21	77	NORMAL	70	CF					
N22	87	NORMAL	60	CM					
N23	90	NORMAL	54	CF					
N24	95	NORMAL	56	CM					
N25	97	NORMAL	73	F					
N26	110	NORMAL	42	CM					
N27	174	NORMAL	18	CF					
N28	178	NORMAL	29	HM					
N29	246	NORMAL	20	OM					
