# Supplementary material for: Cardioprotective role of APIP in myocardial infarction through ADORA2B
Source: Cell Death Dis. 2019 Jul 1;10(7):511. doi: 10.1038/s41419-019-1746-3 (PMC6602929; doi:10.1038/s41419-019-1746-3)
Supplement: Supplementary file 10 — Supplementary figure legends [file 41419_2019_1746_MOESM10_ESM.docx]

**Supplementary figure legends**

**Supplementary Figure 1. Induction of APIP under hypoxia.**

(**a**) Temporal mode of APIP and HIF1α regulation under hypoxic conditions. Cardiomyocytes isolated from neonatal WT mice were exposed to normoxic (Nor) or hypoxic (Hypo) conditions for the indicated times and analyzed by western blotting.

(**b**) Increased levels of APIP protein and mRNA transcript in *APIP^Tg/+^* mice. Heart extracts from WT and *APIP^Tg/+^* mice (2-month-old) were examined by western blotting (*upper*) and real-time RT-PCR analysis (*lower*).

(**c**) Histochemical analysis of paraffin-sections of the hearts from WT and *APIP^Tg/+^* mice. Representative images from immunohistochemical analysis using APIP antibody were prepared with the heart section of WT and *APIP^Tg/+^* mice (4-month-old).

**Supplementary Figure 2. Knockdown of APIP by shRNA in H9c2 cells.**

H9c2 cells were transfected with pSUPER-neo (Mock) or *Apip* shRNAs (sh*Apip* #1 and sh*Apip* #2) for 24 h and total RNAs were analyzed by real-time RT-PCR analysis (*n* = 4, ***P* < 0.01, **P* < 0.05, one-tailed *t* test).

**Supplementary Figure 3. *In situ* PLA assay for endogenous APIP-ADORA2B interaction.**

(**a** and **b**) Intracellular localization of APIP and ADORA2B in the Duolink® *in situ* PLA assay in HeLa cells. Each red dot represents a single protein–protein interaction. Nuclei were stained with DAPI (blue) and representative confocal images are shown. Scale bars, 20 µm (a). Quantification of APIP/ADORA2B PLA signals in HeLa/Mock and HeLa/shAPIP cells. The dot plot represents the number of PLA dots per cell (b).

**Supplementary Figure 4. Mapping of APIP domain interacting with ADORA2B.**

(**a**) Interaction between APIP and ADORA2B or ADORA3. HEK293T cells were transfected with FLAG-APIP and ADORA1, ADORA2A, ADORA2B, or ADORA3 for 24 h and analyzed by immunoprecipitation (IP) assays using HA antibody.

(**b** and **c**) Involvement of APIP N-terminal region in the binding to ADORA2B. HEK293T cells were transfected with ADORA2B-HA and APIP, FLAG-APIPΔN60, FLAG-APIPΔC62, or FLAG-APIPΔ123-180 (b) or APIP, FLAG-APIPΔN20, FLAG-APIP ΔN40, FLAG-APIP-ΔN50, or FLAG-APIP-ΔN60 (c) for 24 h and analyzed by immunoprecipitation (IP) assays using HA antibody.

(**d**) Schematic representation of APIP and its deletion mutants. The binding activities of APIP WT and mutants to ADORA2B are summarized based on the results in Supplementary Figure 3b and c.

(**e**) Involvement of the N-terminal _44_Thr-Gly-Gly_46_ motif of APIP in the binding to ADORA2B. HEK293T cells were transfected with ADORA2B-HA and FLAG-APIP, FLAG-APIP T44A/G45A, or FLAG-APIP T44A/G45A/G46A for 24 h and analyzed by immunoprecipitation (IP) assays using Anti-FLAG M2 Affinity gel.

**Supplementary Figure 5. ADORA2B regulates APIP level**.

(**a**) ADORA2B knockdown decreases APIP level under hypoxia. HeLa cells were transfected with pSUPER-neo or sh*ADORA2B* (sh*A2B*) for 24 h and incubated under hypoxic conditions for 10 h.

(**b**) Elevation of APIP level by ADORA2B agonist in primary cardiomyocytes. Cultured cardiomyocytes were incubated with serum-free medium for 24 h, treated with the indicated concentrations of BAY60-6583 for 10 min, and analyzed by western blotting.

(**c** and **d**) Crucial role of APIP in ADORA2B-mediated HIF1α stabilization and cytoprotection against hypoxic stress. HeLa cells were transfected with ADORA2B, *Apip* shRNA or both ADORA2B and *APIP* shRNA for 24 h, and incubated under the hypoxic condition for 6 h (c) and 36 h (d). Cell lysates were examined by western blotting (c) and cell death were assessed by trypan blue exclusion assay (**P* < 0.05, ***P* < 0.01, ****P* < 0.001, *N.S.*, not significant (*P* > 0.05), one-way ANOVA/Bonferroni) (d).

**Supplementary Figure 6. ADORA2B D296G mutant does not induce the accumulation of HIF1α under hypoxia.**

(**a** and **b**) Asp296 of ADORA2B is important in HIF1α accumulation under hypoxic stress. HeLa (a) and HEK293T (b) cells were transfected with pDEST-HA (-), ADORA2B-HA, or ADORA2B D296G-HA for 24 h, switched to serum-free medium for 24 h, pre-treated with 10 μM BAY60-6583 for 10 min, and then incubated under the hypoxic condition for 3 h. Cell extracts were analyzed by western blotting.

**Supplementary Figure 7. Generation of *Adora2b* D296G knock-in mice.**

(**a**) Scheme for genotyping strategy of *Adora2b* D296G knock-in mice.

(**b**) Schematic illustration of *Adora2b* D296G mutation.

(**c**) *Adora2b* genotyping by genomic PCR. Genomic DNA was extracted from mice of different Adora2b genotypes and subjected to amplification by PCR. PCR products are resolved by agarose gel electrophoresis.

**Supplementary Figure 8. ADORA2B accumulation by APIP is regulated via the lysosomal event.**

(**a** and **b**) ADORA2B destabilization by APIP knockdown is suppressed by bafilomycin A1. HeLa/Mock and HeLa/sh*APIP* cells were treated with 10 nM bafilomycin A1 (BafA1) for 6 h. Cell lysates were analyzed by western blotting (a) and the signals on the blots were quantified by densitometric analysis (**P* < 0.05, *N.S.*, not significant, one-way ANOVA/Bonferroni) (b).

(**c**) ADORA2B destabilization by APIP knockdown is not affected by MG132. HeLa/Mock and HeLa/sh*APIP* cells were treated with 5 μM MG132 for 6 h and analyzed by western blotting.

**Supplementary table legend**

**Supplementary Table 1. Patient and control groups used for the gene expression analysis.**

We examine the expression levels of *APIP* and *ADORA2B* mRNAs in cardiac tissues from 29 healthy adults (control group) and 18 patients suffering from ischemic heart disease (IHD), congestive heart failure (CHF), dilated cardiomyopathy (DCM), or heart failure (HF) using RT-PCR.
